# Photosensitive Control and Network Synchronization of Chemical Oscillators

**DOI:** 10.3390/e26060475

**Published:** 2024-05-30

**Authors:** Alejandro Carballosa, Ana I. Gomez-Varela, Carmen Bao-Varela, Maria Teresa Flores-Arias, Alberto P. Muñuzuri

**Affiliations:** 1 Laboratoire de Physique Théorique et Modélisation, CY Cergy Paris Université, CNRS, UMR 8089, 95302 Cergy-Pontoise, France; ac.carballosa@gmail.com; 2 Group of Nonlinear Physics, Department of Physics, University of Santiago de Compostela, E15782 Santiago de Compostela, Spain; 3 Galician Center for Mathematical Research and Technology (CITMAga), E15782 Santiago de Compostela, Spain; 4 Photonics4Life Research Group, Applied Physics Department, Institute of Materials (iMATUS), Universidade de Santiago de Compostela, Campus Vida, E15782 Santiago de Compostela, Spain; anaisabel.gomez@usc.es (A.I.G.-V.); carmen.bao@usc.es (C.B.-V.); maite.flores@usc.es (M.T.F.-A.)

**Keywords:** complex networks, Belousov-Zhabotinsky reaction, microreactors, SLIPAA technique, synchronization, photosensitivity, oscillatory behavior

## Abstract

The Belousov–Zhabotinsky (BZ) reaction has long been a paradigmatic system for studying chemical oscillations. Here, we experimentally studied the synchronization control within photochemically coupled star networks of BZ oscillators. Experiments were carried out in wells performed in soda-lime glass constructed using novel laser technologies. Utilizing the inherent oscillatory nature of the BZ reaction, we engineered a star network of oscillators interconnected through photochemical inhibitory coupling. Furthermore, the experimental setup presented here could be extrapolated to more complex network architectures with both excitatory and inhibitory couplings, contributing to the fundamental understanding of synchronization in complex systems.

## 1. Introduction

One of the main goals of understanding the natural mechanisms that govern synchronization processes is to be able to control them. The malfunctioning of biological rhythms in the heart or the brain may cause severe damage in the form of arrhythmias [1,2] or epileptic seizures [3,4], thus the ability to counter these anomalous and irregular behaviors is of great importance [5]. In this sense, the synchronization of nonlinear systems became a very important topic in the 90s as it was proven that chaos itself could be controlled and driven by external sources [6,7], with potential applications to communications. This premise triggered an extensive research in chaos synchronization (see [8] for a review) that involved a surge of many experimental studies in lasers and circuits, some also aiming to develop secure communication schemes [9,10,11,12,13]. Later on, this idea of targeting and steering the dynamics of a slave system by synchronizing them to the dynamics of a master one was extrapolated to any type of generic dynamical system in complex networks [14,15,16]. In the context of chemical systems, there is also a large history of synchronization studies based on the oscillating Belousov–Zhabotinsky (BZ) reaction [17]. Starting from the simplest setups of just a very few number of oscillators coupled diffusively [18,19,20], where synchronous behaviors such as in and out of phase entrainment or oscillation death could already be studied, soon, more complicated ones appeared that could account for larger populations of oscillating particles [21] or even spatial arrays of micro-oscillating units [22]. With the photosensitive version of the BZ reaction, it was later observed that these synchronous dynamics and other spatiotemporal ones such as spiral waves and chimera states could also be driven by periodic external light sources [23,24,25]. Furthermore and just very recently, a computational programmable machine has been developed using spatial arrays of BZ interconnected cells, showing that this computer analog can exhibit cellular automata rules and solve combinatorial optimization problems [26]. 

In this work, we aimed to contribute to the design of control experiments by proposing a novel experimental setup where the dynamics of chemical oscillators based on the BZ reaction is individually controlled through light pulses. The wells for the reactions were constructed using the subaquatic indirect laser-induced plasma-assisted ablation (SLIPAA) procedure in soda-lime materials, a new glass processing technique patented in 2021 by some of the authors [27]. The setups built in this way are compatible with high acidic reactions (such as the one used along this manuscript) as well as with biological systems opening, thus, presenting interesting possibilities. A direct application of this setup, as we shall see later, is the possibility of building complex network structures of chemical oscillators. In this direction, very recent experiments have followed this line of work by examining star-networks, discovering intricate connections between the node degree and the dynamical regime [28], and novel synchronization modes between the center and peripheral nodes [29]. In the first experiment, micro water droplets loaded with BZ dynamics were suspended into star-like silicon structures of different connectivities, where nodes of the network connect via micro-fluidic arms. In the latter, photosensitive catalyst-loaded beads are immersed in a BZ catalyst-free solution (like the ones used in [23,24]) and are then connected by short light pulses that are triggered any time one of the bead particles oscillate. A particular consideration explored in this latter experiment was that the central/master node has a significantly larger natural period than the peripheral ones, which oscillate much faster, which induces phase delays in the peripheral oscillators that make them synchronize in the form of one whole cluster or two clusters of different populations. In contrast to these two, our experimental setup considered features from both approaches and considered silica gels of 2 mm of diameter rather than resin microbeads of 200 µm. This makes it easier to resolve the individual oscillations of a larger number of oscillators arranged closely together in the previously mentioned wells, in such a form that the oscillations are tracked separately while there is not a direct exchange of chemical information between them (the wells are not connected via micro-fluidic channels as in [28]). Furthermore, our oscillators were prepared under similar conditions, so there were no significant distinctions between them. Following [29], the connection between oscillators was carried out through the photosensitive mechanism via short pulses related to the oscillator firings in such a way that we can consider directional links between the nodes. 

The rest of this paper is structured as follows. Section 2 describes the experimental setup and the methodology used in our control experiments. Section 3 presents the experimental results, and Section 4 reports a summary and discussion of the findings.

## 2. Methods

### 2.1. Fabrication of the Reactors Using the SLIPAA Technique

The reactor was designed as a two-dimensional array or small wells (2 mm in diameter and 2 mm depth) separated by a distance large enough to prevent direct interaction between each other. Glass is one of the more suitable materials used for this purpose due to its low capacity to react with the chemical components used. However, structuring glass poses challenges. In recent decades, lasers have emerged as an alternative tool for glass machining. Here, a Q-Switched Nd:YVO_4_ laser (Rofin; Plymouth, MI, USA), operating at 1064 nm and a pulse duration of 20 ns combined with a galvanometer system for steering the output laser beam, was used for this end. A flat-field lens with a focal length of 160 mm provided a uniform irradiance distribution on the glass substrate over a working area of 120 × 120 mm^2^. To achieve the desired well structure, a thin water layer was added between a metallic target and the glass substrate. When the laser beam is focused on the metallic target, after going through the glass substrate and the water layer, the glass is processed as a result of a combination of the ablation mechanism, the shock waves, and the cavitation bubbles generated. 

**Figure 1:**
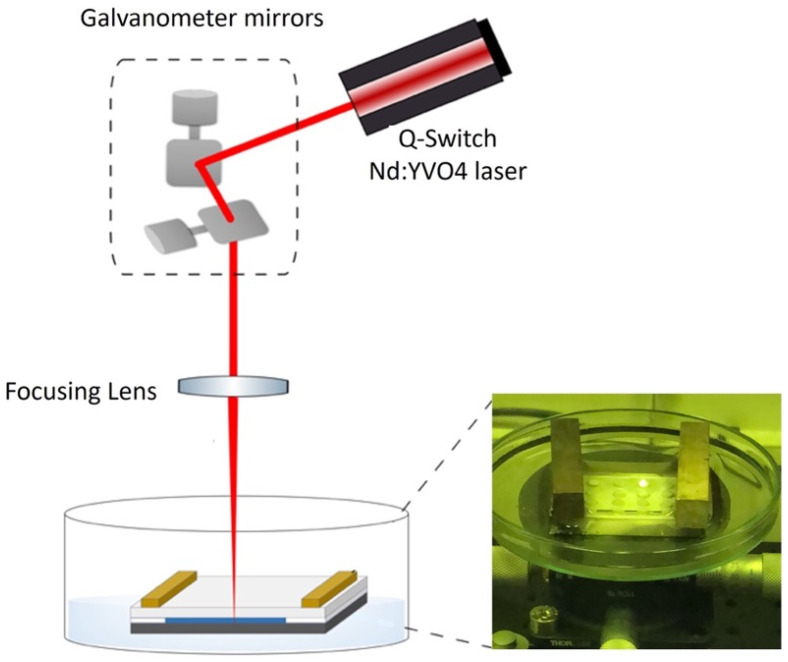
Setup of the SLIPAA fabrication technique. The right side of the figure shows a photograph of the wells during the fabrication process. Note that a white colloidal suspension was generated during the ablation process of the structures.

Figure 1 shows the setup used to fabricate the well array in this work. Ablation was conducted under static flow conditions with a deionized water layer with a thickness of 145 μm (at room temperature) situated between the glass substrate and the metal target, assuring in this way that the thermal effects over the glass were minimized [27]. Note that the depth and optical properties of the ablated structures depend on a set of laser parameters (wavelength, pulse duration, repetition rate) and experimental conditions (laser scanning speed, liquid layer absorption and so on) that must be optimized to achieve high quality structures without cracks and chips. For this particular application, an average laser power of 4.92 W at a repetition rate of 10 kHz was used, combined with a beam scanning speed of 200 mm/s. After 30 laser passes, wells with a good rectangular cross-section were achieved and good to excellent optical quality in terms of transparency, given the type of experiments in mind. The metallic sheets used as targets are known as “Hardened Spring Steel W.-Nr. 1.1274” (H + S Präzisionsfolien GmbH, Pirk, Germany) with a carbon content of over 1% and the soda-lime substrates with a low absorbance at the 1064 nm wavelength were acquired from a local supplier. 

With the fabrication method described above, wells of different diameters and depths as well as more complex structures can be fabricated. For instance, wells with a 4 mm depth can be obtained by refocusing the laser beam and washing away the ablation debris generated during SLIPAA processing. The key factor here is to avoid unintended displacement of the substrate and the generation of air bubbles that may be trapped inside the structures, thereby affecting the quality of the final structure [27]. In fact, several configurations were produced, although, finally, the experiments shown here were conducted with the device described. 

### 2.2. Experimental Setup for Control Experiments

**Figure 2:**
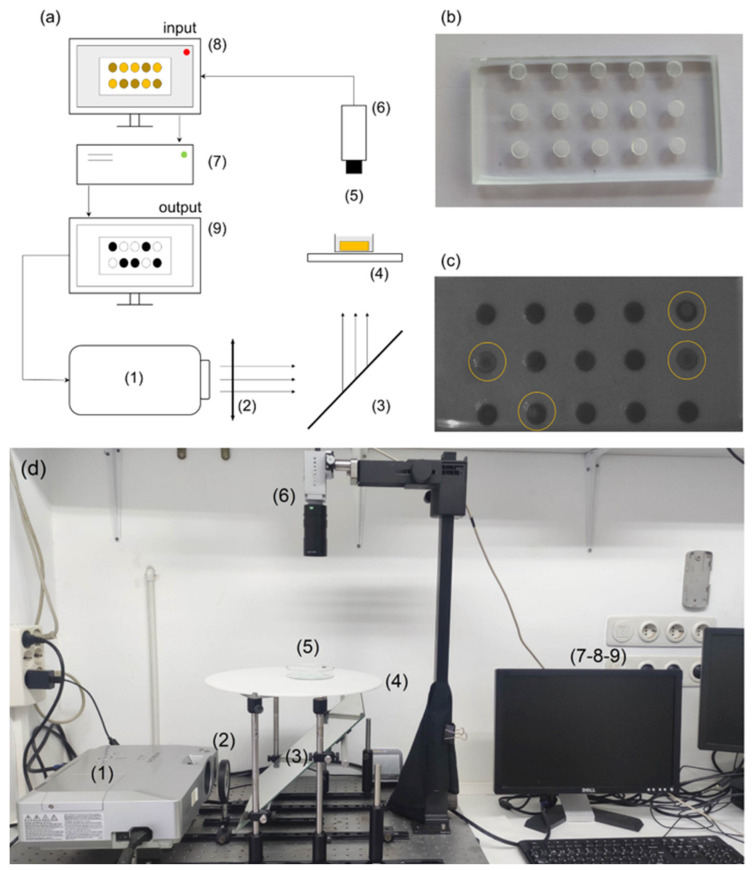
(**a**) Schematic representation of the experimental setup. (1) video projector, (2) collimating lens, (3) reflexive mirror at 45°, (4) light diffusor, (5) Petri dish with the reactors and BZ solution, (6) CCD camera, (7) CPU, (8,9) monitors to control and analysis of both the experiment and the projected illumination field. (**b**) Glass reactors fabricated via SLIPAA techniques. (**c**) Snapshot of an ongoing experiment where each dark circle is a BZ oscillating reactor. The yellow circles indicate ongoing oscillations. (**d**) Photograph of the experimental setup.

The experimental setup for the synchronization and control experiments is shown schematically in panel (a) of Figure 2, with photographs of the empty glass reactor in panel (b), the reactor during an ongoing experiment (c), and the actual experimental setup (d). First, we used a Hitachi CP-X300 video projector (marked with 1 in Figure 2a) to illuminate the reaction from below. This device allowed us to project illumination fields of different intensities with eight-bit grayscale between 0 and 255, not only varying in space, but also in time. The projected image first goes through a collimating lens (2) in order to correctly focus the image on the reflective mirror (3). Before reaching the Petri dish that contains the reactor and the reaction (5), the illumination goes through a diffusor (4). The purpose behind this is, on the one hand, to equalize the spatial inhomogeneities produced by the bulb of the video projector, and, on the other hand, to enhance the contrast of the transmitted light monitored through a PixeLink CCD video camera (6).

The BZ reaction takes place in a Petri dish placed on top of the diffusor (5). For this, we first filled the holes of the glass reactor shown in panel (b) with a silica gel loaded with the ruthenium catalyst [30]. We considered a silica gel for the substrate of our oscillators as they are easy and quick to fabricate, but especially because they allowed us to precisely monitor and control the individual oscillations of many oscillators under the CCD camera angle. Furthermore, it is a setup that has been proven to be very efficient in the photosensitive control of waves and other oscillating phenomena in the BZ reaction [31]. Loading the metal-ion catalyst in a gel allows it to remain fixed, so the oscillations occur within the surface of the gel, as can be seen in panel (c). The wells in the reactor were made using the technique described in the previous section [27,32]. Because of the small diameter of the wells (∼2 mm), the oscillation wave in the reactor covered the surface almost instantaneously, avoiding the emergence of multiple wave centers and reducing the possibility of emerging spiral waves. Additionally, the reactors were separated within a distance of 3 mm, which was cleaned before initiating the experiment to avoid interactions between neighboring wells. After the reactor was prepared, we placed it inside the Petri dish and covered it with the catalyst-free solution of the BZ reaction until it was completely submerged (see Supplementary Materials for details on the chemical compositions). Note that with this technique, we can easily scale up our experiments by increasing the number of oscillators in the networks. Here, we kept the number reduced in order to see the details of the synchronization mechanism involved.

**Figure 3:**
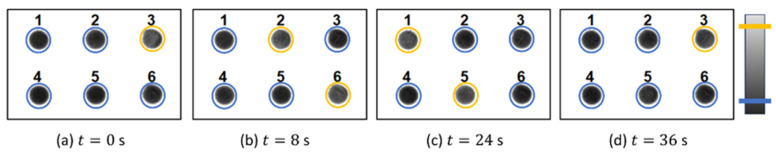
Consecutive snapshots of six uncoupled BZ reactors (wells) recorded in an experiment. The oscillations can be seen from the contrast between black and gray. As this contrast can be difficult to grasp, we added a yellow circle as a visual aid that means that the oscillator is firing, while a blue one means that it is resting (see color bar in the side). At t=0, only oscillator 3 is activated. At t=8 s, oscillators 2 and 6 fire together, while at t=24 s, it is the turn of oscillators 1 and 5. Finally at t=36 s, oscillator 3 fires again, and the cycle repeats.

The oscillations in the reactor are recorded through the CCD video camera (6) and are monitored (7) and recorded in a CPU (8), where the frames are analyzed in real-time to measure whether an oscillator has just fired or not. Simultaneously, the computer is connected to the projector (1) to send spatio-temporal modulations of the light intensity (9) at the exact positions of the small wells, establishing a feedback loop that modifies the oscillating period of each well. With this setup, we can record up to N=100 oscillating reactors and, through real-time monitoring, send illumination pulses that can either excite or inhibit oscillations in other wells. In Figure 3, we show an example of a series of recorded snapshots where six uncoupled reactors (wells) are firing at different times. The firing of the oscillator can be noticeable in the grayscale, as the oscillators pass from black to light gray (highlighted with a visual aid in the color bar).

We considered the photosensitive version of the BZ reaction that is catalyzed by the $\mathrm{Ru}{\left(\mathrm{bpy}\right)}_{3}^{2+}$ complex, which has been extensively analyzed in previous communications [33,34]. This catalyst is known to be activated by visible light, becoming a powerful reducing agent that directly intervenes in bromomalonic acid production, inducing the additional production of bromide ions that inhibits the reaction, and thus increases the period of oscillation. If the global amount of light reaching the reaction is reduced, then, the effect is the opposite and the period is reduced.

### 2.3. Periodic Pulses and Coupled Networks

**Figure 4:**
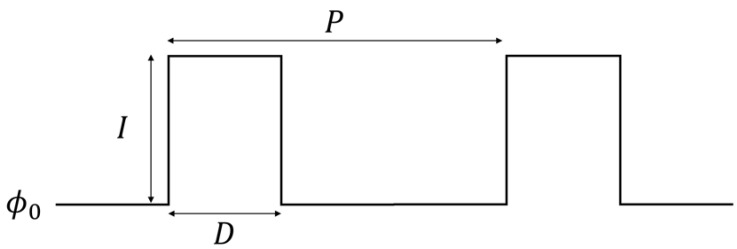
Square signal of period P, size D, and amplitude I used for the periodic control experiments. Note that this pulse actually results in a total light intensity experienced by the oscillators of $\phi =\phi_{0}+I$.

Our first attempt to control synchronization consisted of homogeneously stimulating the oscillators with periodic pulses in order to adapt their natural frequencies to the signal. For this purpose, we designed a square signal of period P and time duration D like the one drawn in Figure 4. The total light intensity ϕ of the signal takes into account the background illumination $\phi_{0}$ and the height or amplitude of the signal I (i.e., $\phi =\phi_{0}+I)$. Note that this amplitude can be either positive, in order to induce photoinhibition, or negative, to induce the photoexcitation of oscillations. A preliminary study of the system under these pulses will allow us, on the one hand, to better characterize the system studying the oscillator’s response to light stimulations.

On the other hand, by monitoring in real-time the state of each oscillator, we can send light pulses between the oscillators every time they fire. That is, if we establish a connection between oscillators i and j, then every time oscillator i fires, the square pulse of intensity I and duration D is sent to oscillator j, and vice versa. In this way, generating an arbitrary adjacency matrix $A_{ij}$ where each element denotes whether a connection exists between nodes i and j, we can create any kind of network of oscillators.

To measure synchronization in these types of chemical oscillators, the usual approach is to apply a Hilbert transform to the oscillator’s recorded amplitude in order to obtain a phase variable for each one, and then use the Kuramoto order parameter to measure synchronicity [21,35]. However, the error in the Kuramoto parameter scales with the number of oscillators as $\sim N^{-\frac{1}{2}}$, so for a low number of oscillators as we have, it does not provide an accurate portrait. Thus, we propose the following estimator in order to compare the observations found within our experiment. Finding the location in time of the oscillator firings, we subdivided the data series into equal fragments given by the average period of oscillations T, and for each fragment, we measured the standard deviation σ of the firing times. In this way, if all the oscillations are firing together, σ=0. We will measure these deviations before and after the control experiment, $\sigma_{b}$ and $\sigma_{f}$, so we can define an order parameter ρ as:

(1) $$\rho =1-\frac{\sigma_{f}}{\sigma_{b}}$$

which depends on both the final standard deviation of the firings and on how much the synchronization was improved after the experiment. This serves us as a way to normalize each experiment within its own experimental conditions while at the same time having an estimator that can range between 0 and 1. Note that we were unable to track the state of excitation for each oscillator during the forcing interval as the illumination was modified during the process, and thus, the excitation state was not observable. 

## 3. Results

### 3.1. Characterization of the Experimental Setup

Small differences in the preparation of the gels may induce small heterogeneities in the oscillators, which are directly related with the concentration of catalyst in each well. This translates in standard deviations of the natural periods of around σ(T )≃1−2 s in general, as can be grasped from Figure S1 of the Supplementary Materials. There we report a representative distribution of oscillation periods centered around the mean value and measured at the beginning of an experiment with N=28 oscillators. On the other hand, the effect of light reduces the excitability (due to the extra production of bromide ion by the light-activated catalyst [33]) of the system, and thus the oscillation period is increased [36], while higher temperatures induce a higher frequency of oscillations [37,38,39]. Therefore, we kept the experiments in a dark room with close to zero ambient light and kept the room temperature constant (between 18–22 °C) with the aid of an AC system. The periods of the oscillators also decayed with time, as the system is a batch reactor and the chemicals are consumed. We represented this dependence in the Supplementary Materials (Figure S2).

**Figure 5:**
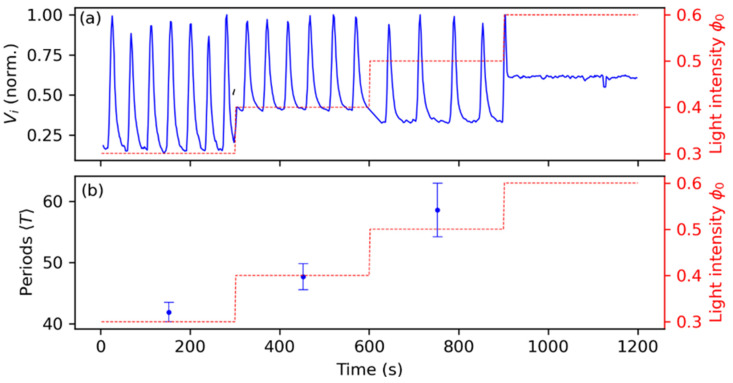
Effects of the homogeneous background light illumination $\phi_{0}$ on the chemical oscillators. Panel (**a**) reports the recorded pixel value Vi of a single oscillator, showing the oscillations in the metal-ion catalyst, while panel (**b**) shows the average period and standard deviation of the whole ensemble of N = 20 oscillators.

The effect of light illumination in the oscillators is reported in Figure 5, with panel (a) showing the recorded pixel color values $V_{i}$ in time for one single oscillator (rescaled in a 0 to 1 range), and panel (b) showing the periods of the ensemble (composed of N = 20 oscillators) for different values of the background intensity $\phi_{0},$ which is characterized by 0 (total darkness) to 1 (total white). Starting from $\phi_{0}=0.3$, it can be seen that each time we increased the light intensity by $\Delta \phi_{0}=0.1$, there was an increase in the periods of ΔT=5.8 from $\phi_{0}=0.3$ to $\phi_{0}=0.4$ and of ΔT=10.93 from $\phi_{0}=0.4$ to $\phi_{0}=0.5$. From there on, as we can see in panel (a), the oscillators enter the excitatory state and only a few oscillators remain active because of the aforementioned heterogeneities.

### 3.2. Synchronization Control by Means of Periodic Pulses

With the periodic square pulses described in the Methods (see Figure 4), we examined the effect of the time duration D and light intensity $\phi =\phi_{0}+I$ of the signal in the overall synchronization of the oscillators. Regarding the period *P* of the signals, prior to each experiment, we fixed it close to the current natural period of the oscillators, as the final aim of the experiment was that the signals are sent by the oscillators themselves. We noted that as the period of the oscillators decay as the reactants are consumed in the reactors, *P* must be adjusted for each experiment (see Figure S2 of the Supplementary Materials for further information). Therefore, we focused on entraining the oscillators to periods close to their natural ones. For every experiment, the background illumination was also fixed at $\phi_{0}=0.4$ in order to ensure good visibility of the oscillations.

**Figure 6:**
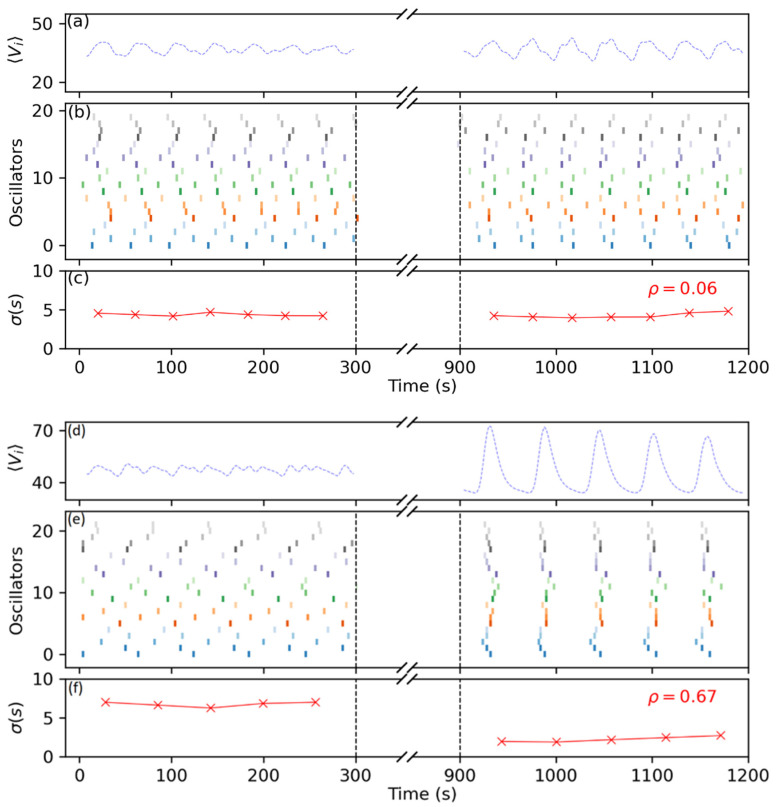
Collective oscillations before and after our control experiments with periodic square pulses, for (**a**–**c**) low pulse intensity ϕ=0.5, and (**d**–**f**) high pulse intensity ϕ=1.0. The skipped area in between the black dashed lines represents the control region where we applied the square signal. Panels (**a**) and (**c**) correspond to the average values of the pixel intensity of the whole ensemble $V_{i}$, while panels (**b**) and (**e**) show the firings in time of each oscillator (in colors). Panels (**c**) and (**f**) correspond to the standard deviation σ between the firing times of the oscillators, measured in seconds. For both panels, N = 21, background intensity was set to $\phi_{0}=0.4$ and pulse duration was set to D=10.

Figure 6 shows the outcome of two control experiments with different levels of the pulse amplitude ϕ, namely ϕ=0.5 (panels a–c) and ϕ=1.0 (panels (d,e)), and a pulse duration of D=10 s. During the control part of the experiments, we let the oscillators run freely for about 5 min (300 s) and then applied the control strategy for the next 10 min. This is highlighted in the figure by the black dashed lines and the broken axis. During this part, accurately recording the oscillations is difficult as the illumination square pulses shine very brightly and saturate the CCD camera, also masking the oscillations themselves in the process. Therefore, in order to measure the state of the system after having applied the control procedure, we let the oscillators run free again (in the absence of periodic light forcing) under the same illumination conditions as those used previously to the entrainment periodic pulses and recorded for the next 5 to 10 min. Panels (a) and (d) show the average recorded pixel values $V_{i}$ of all the oscillators in the ensemble, shown as a preliminary measure of the system’s coherence. Panels (b) and (e) show the individual firings of the oscillators in the form of a raster plot, where each row is a different oscillator, and the firings correspond to the colored vertical bars. For the case of ϕ=0.5, we can see before the control section that although the oscillators fired somehow next to each other, the overall levels of synchronization were quite low and the averaged signal was noisy. This was confirmed by the dispersion in the firings, as shown in panel (c), where σ∼5 s. After the control experiment, we could see that this dispersion had not decreased much, but at least the oscillators fired closer to each other in a more ordered fashion, as can be grasped from the distorted oscillations in the global averaged signal (panel (a)). In this case, the order parameter ρ, computed as described in Equation (1), using the average of the dispersions before and after the experiment, returned a value of ρ=0.06. This low value of the order parameter mainly reflects the lack of control in the firing dispersions, as the difference between both sides was almost null.

On the contrary, in the experiment with high levels of light intensity with ϕ=1.0 (panels d–f), the contrast between the two recorded moments was a lot clearer, with the oscillators firing almost simultaneously and the global averaged signal being well-defined. This was also reflected by the firing dispersions, now being of the order σ=1.8 s, and the order parameter returned a value of ρ=0.67. One of our choices to define ρ as we did is illustrated by contrasting these two cases. In panel (c), the system was slightly more ordered after the control experiment because it was not that disordered from the beginning. On the other hand, we can see from panel (f) that initial dispersions in the firings can be largely corrected if the pulse is strong enough. Therefore, we decided to normalize $\sigma_{f}$ over $\sigma_{b}$ in order to take into account this feature, as described in the previous section. Furthermore, in the absence of stimuli and as can be grasped from panel (e), the oscillators quickly relaxed to their natural unsynchronized frequencies after a few oscillations, and therefore we limited our measuring window to the first five minutes after the experiment.

**Figure 7:**
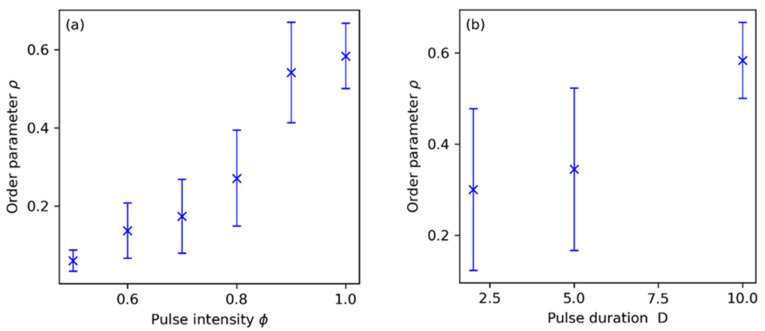
(**a**) Overall levels of synchronization ρ of the control experiments as a function of the pulse intensity ϕ, and (**b**) as a function of the pulse duration D in seconds. For panel (**a**), pulse duration is fixed at D =10, while in panel (**b**) pulse intensity is fixed at ϕ=1.0. In both panels, error bars represent the standard deviation of our synchronization measures between different realizations of the experiment, with at least 5–10 experiments for each point and 20–25 oscillators in each experiment.

We further characterized our system by repeatedly measuring the parameter ρ for different values of the maximum light intensity ϕ and pulse duration D, as shown in panels (a) and (b) in Figure 7, respectively. For this purpose, we repeated several experiments under the same conditions and measured the firing dispersions σ as in the previous cases. We found that between ϕ=0.5 and ϕ=1.0 (see panel (a), with D=10), there was quite a spectrum of order levels that increased monotonically with ϕ. On the other hand, with shorter pulses of just D=2 s (see panel (b), with now ϕ fixed to 1), the variation from experiment to experiment was high and we never found synchronization values higher than ρ = 0.5. For pulses of D = 5 s, the situation was quite similar, with only slight increases in the order parameter. For D=10 s, we recovered the synchronization levels found in Figure 6.

### 3.3. Networks of Coupled Oscillators

**Figure 8:**
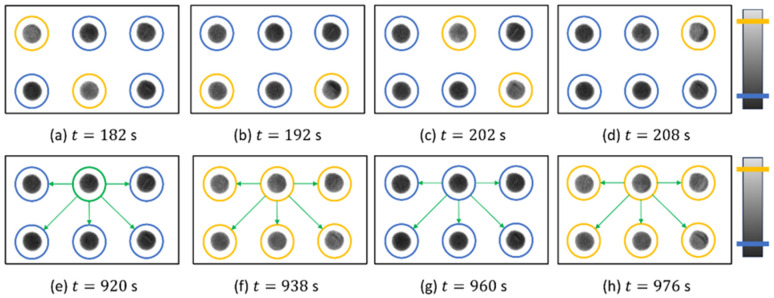
Consecutive snapshots of six BZ reactors recorded before (**top row**, **a**–**d**) and after (**lower row**, **e**–**h**) an experiment with coupled-star networks. As in Figure 3, a blue circle means that the oscillator is resting while a yellow circle shows that the oscillator is firing. Panel (**e**) explicitly shows the distribution of the start network topology, with the oscillator circled in green acting as the center node, sending light pulses to the ones next to it after firing. After this panel, the previous color coding is recovered, but the network structure is maintained. At t=938 s and t=976 s, we can see all the oscillators firing together with a common period of 〈T〉=38 s. The time snapshots of these panels correspond to the experiment shown afterward in Figure 9.

In this section, we considered a second application of the setup: building a network of self-regulated chemical oscillators via light coupling. Here, we present a first-order approximation to the problem by considering a star network such as the one shown in Figure 8e. There, we show the results of an experiment where there is one master oscillator (green, top center) that sends light pulses to its peripheral connections (blue) every time it fires.

**Figure 9:**
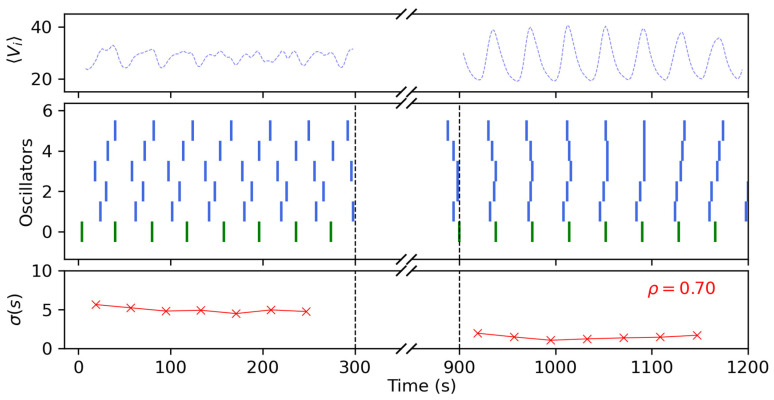
Collective oscillations before and after our control experiments with a star network coupling, following the disposition of Figure 8. Again, the area between the black dashed lines represents the control region where we applied the coupling. The green oscillator corresponds to the central one, the master, while the blue ones are the peripheral oscillators that receive the pulses. The dashed blue line corresponds to the global average recorded signal of all oscillators. For this experiment, N = 6, the background intensity was set to $\phi_{0}=0.4$, the pulse coupling had a duration of D = 10 s, and maximum light intensity of ϕ=1.0.

These snapshots recorded before (top row, panels (a–d)) and after (lower row, (e–h) the control experiment show that the network of six oscillators that was initially asynchronous, becomes perfectly synchronized, firing in unison at t = 938 and t = 976 s, thus showing a common period of 〈T〉=38 s. A more complete description of this experiment is shown in Figure 9, following the same schematics as in the previous sub-section. From the results obtained there, for this experiment, we selected the optimal combination of control parameters that could grant synchronization, which were ϕ=1.0 and D=10. Here, we can better grasp how after the coupling, the peripheral oscillators underwent a series of adaptations to the master’s signals, eventually firing all together.

**Figure 10:**
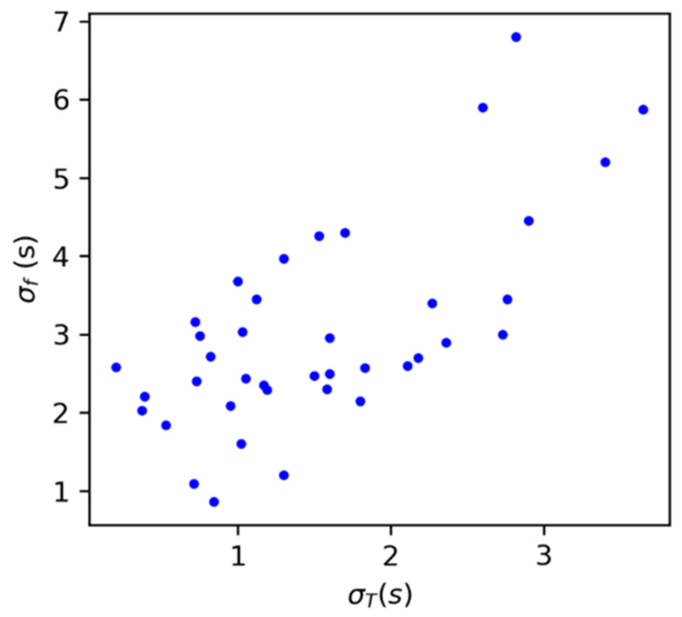
Dispersion in the firing of the oscillators after the control experiment σ_f_ against the dispersion in the natural periods between the peripheral oscillators and the central one,$\;\sigma_{T}$. Each point represents a different experiment under the same coupling conditions, $\phi_{0}=0.4$, ϕ=1.0, and D = 10.

As we previously commented, each experiment was endowed with a certain degree of noise related to the small heterogeneities in the natural periods of the oscillators. We observed in our coupled networks that to obtain a synchronized group, the natural periods of the peripheral oscillators must not differ too much from that of the central one. This is a general result from the synchronization phenomena obtained from the Kuramoto model that oscillators with closer natural frequencies are the ones to couple first, while those with larger frequencies will be absorbed by the main cluster depending on the intensity of the coupling strength K [35]. We report this in Figure 10. For each control experiment, we measured the final dispersion in the firings $\sigma_{f}$, and the dispersion in the natural periods of the peripheral oscillators from the central one,

(2) $$\sigma_{T}=\sqrt{\frac{1}{n}\overset{n}{\underset{j}{\sum }}{\left(T_{j}-T_{c}\right)}^{2}}$$

where n is the number of peripheral oscillators, $T_{j}$ is their natural period before the control experiment, and $T_{c}$ is the natural period of the central oscillator before the experiment. Attending to these measures, we can grasp a monotonical increasing tendency between the two, finding that if the dispersion in the natural periods is larger than $\sigma_{T}>3\;$s, then the peripheral oscillators are not able to adapt to the central one. For lower values of $\sigma_{T}$, we noticed an important degree of noise in the system by looking at the difference in the degree of synchronization between experiments with dispersions lower than $\sigma_{T}<1$ s. Here, we found several experiments with low initial dispersion that produced totally different outcomes. 

## 4. Conclusions

Here we presented a suitable experimental setup to study synchronization properties in networks of chemically coupled oscillators. For the experimental design, a series of glass reactors with small wells carved with the SLIPAA technique were prepared explicitly for this application. The technique used is of interest to other chemical and biological studies. Note that the materials used are compatible with both strong acidic reactions (such as BZ) as well as biological systems.

We first approached the problem from the perspective of control, considering a periodic inhibitive signal that could force the oscillators to fire in unison. From the first round of experiments presented here, we determined a set of optimal parameters for the square signal (namely the light intensity ϕ and the pulse duration D) that best allowed us to control the firing of the oscillators. We assessed this by measuring the standard deviations between the firings of the oscillators, finding that they were minimal for the maximum level of light intensity ϕ=1 and for a pulse duration of D=10 s. For smaller values of both quantities, we saw that in each case, some levels of control were exerted on the oscillators, although the overall levels of synchronization were not perfect. This was reflected by the presence of a small clusters of synchronized activity, whose population increased with increasing levels of intensity ϕ, in a sort of partial synchronous state. This is reflected in Figure 7b and is most likely related to the dispersion of natural periods in contrast to the control signal, as we also observed for the networks in Figure 10. On the other hand, and probably also related to the mismatching of periods between the oscillators and the control signal, the presence of two clusters in the opposing phase but similar periods T within this system was also observed in some experiments. An example of this behavior is shown in Figure S3 of the Supplementary Materials. However, this did not occur as a rule, and therefore it was difficult to measure their appearance, or the necessary conditions to trigger them. Therefore, its investigation is left for future work.

We note here that there is also another mechanism to control the firing of the oscillators that is possible within this setup, but that was not shown here. This would consist of using negative values in the square pulse amplitude I, which should excite the oscillators to fire earlier. Its practical implementation requires us to consider some level of illumination as part of the basal state that can be reduced when a negative pulse is received (increasing the excitability of the oscillator and thus reducing its oscillation period). This mechanism can be addressed by using totally different initial concentrations of the BZ reaction; therefore we also leave this mechanism for future work. Being able to use both mechanisms of photoinhibition, together with photoexcitation, would be an optimal scheme for the network couplings.

Moving on with the experiments with pulse-coupled networks, we proved the validity of our setup and compatibility with the chemical reactions used. The example considered a directed star network, which is an over simplification of directed couplings found in nature (particularly in coupled neurons), thus providing us with a well-controlled system to analyze this complicated phenomenon. Here, a central oscillator sent the square pulsed signals to its connections whenever it fired, where we found that these networks were able to self-regulate and adapt if the dispersion of the natural periods of the peripheral oscillators was less than 2 s. Depending on the initial conditions of the oscillators, we observed either full synchronization or clustering. This promising result opens the way for future investigations considering autonomous and more complex networks.

## Data Availability

The data presented in this study are available on request from the corresponding author.

## Supplementary Materials

The following supporting information can be downloaded at: https://www.mdpi.com/article/10.3390/e26060475/s1, Table S1: Recipe for the solution of 5 ml of silica gel loaded with Ruthenium catalyst. Table S2: Recipe for the solution of 20 mL of catalyst free BZ. Figure S1: Distribution of the oscillation periods at an initial moment of one sample experiment. Figure S2: Decay of the oscillation periods in time. Figure S3: Network experiment where two clusters at different phases but similar frequency can be appreciated highlighted with black and red squares.
