# Supplementary material for: Photosensitive Control and Network Synchronization of Chemical Oscillators
Source: Entropy (Basel). 2024 May 30;26(6):475. doi: 10.3390/e26060475 (PMC11203354; doi:10.3390/e26060475)
Supplement: Supplementary file 1 [file entropy-26-00475-s001.zip › entropy-3006619-supplementary.pdf]

Supplementary Information.

Photosensitive control and network synchronization of chemical oscillators

Alejandro Carballosa [1,2,3], Ana I. Gomez-Varela [4], Carmen Bao-Varela [4], Maria Teresa Flores-Arias [4] and Alberto P. Muñuzuri [2,3,*]

[1]Laboratoire de Physique Théorique et Modélisation, CY Cergy Paris Université, CNRS, UMR 8089, 95302 Cergy-Pontoise, France
[2]Group of Nonlinear Physics, Department of Physics, University of Santiago de Compostela, E15782 Santiago de Compostela, Spain

[3]Galician Center for Mathematical Research and Technology (CITMAga), E15782 Santiago de Compostela, Spain

[4] Photonics4Life Research Group, Applied Physics Department, Institute of Materials (iMATUS), Universidade de Santiago de Compostela, Campus Vida, E15782 Santiago de Compostela, Spain

S1. Chemical recipes and elaboration of the Belousov-Zhabotinsky reaction.

The recipe for making 5 ml of gel is indicated in table S1. In a small beaker with a magnetic stirrer inside, the reactants are added in the same order as listed in the recipe. Right after the preparation and before the gelation takes place, the solution is absorbed with the help of a pipette and injected swiftly in each small well until the desired number of micro-reactors are prepared. After a while, the solution gels within each well and the reactor is washed and cleaned with distilled water to remove rests between the micro-reactors.

Quantity	Compound
3,3 ml	$15\%\ \mathrm{NaSiO_3}$
0,62 ml	$\mathrm{H_2O}$
0,150 ml	$22.3\ m\mathrm{M}\ \mathrm{Ru(bpy)^{2+}}$
0,93 ml	$4\mathrm{M}\ \mathrm{H_2SO_4}$

Table S1. Recipe for the solution of 5 ml of silica gel loaded with Ruthenium catalyst

The solution for the free-catalyst Belousov-Zhabotinsky reaction is prepared according to table S2 prior to each experiment and after the silica gels are ready. The stock solutions involved are prepared with distilled water and with reactants commercially available from the SIGMA firm. Namely, we have used, in order of addition: sulfuric acid $\mathrm{H_2SO_4}$, sodium bromide NaBr, malonic acid MA and sodium bromate $\mathrm{NaBrO_3}$. Once it is ready, the solution is pored onto the Petri disc that

contains the reactor, until the latter is smoothly and totally submerged. Thus, the ammount of solution prepared should be enough as to cover the whole reactor.

Quantity	Compound
2,66 ml	H_2O
3,44 ml	$1M\ H_2SO_4$
4,46 ml	$1M$ NaBr
1,67 ml	$1M$ MA
7,77 ml	$1M\ NaBrO_3$

Table S2. Recipe for the solution of 20 ml of catalyst free BZ

S2. Additional experimental results

As mentioned in the main text, small differences in the preparation of the gels may induce small heterogeneities in the oscillators, which are directly related with the concentration of catalyst in each well. Thus, we expect a heterogeneous distribution of oscillatory periods for each experiment that results in a standard deviations of the natural periods of around $\sigma(T) \simeq 1-2$ seconds. One example of this heterogeneity is reported below in Figure S1, for an ensemble of $N = 28$ oscillators.

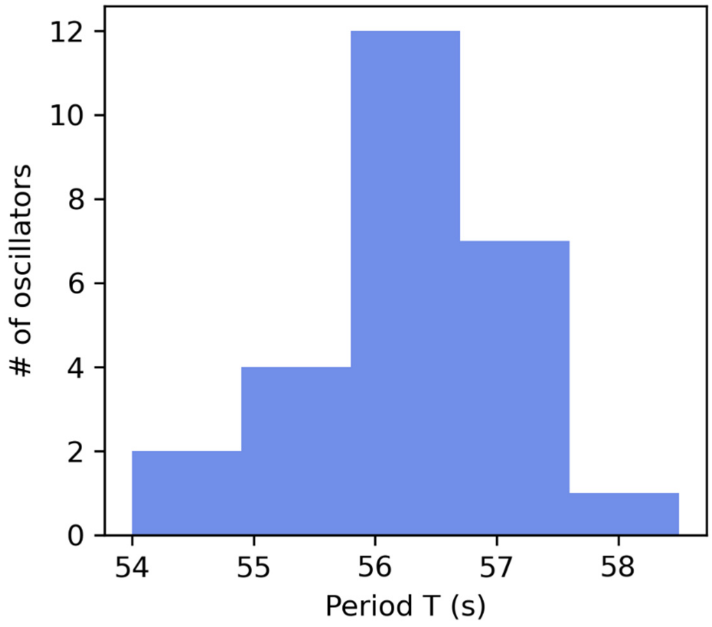

Figure S1: Distribution of the oscillation periods at an initial moment of one sample experiment. The total number of oscillators in this case is of $N = 28$. Here, the average across the ensemble is $\langle T \rangle = 56.33 \pm 0.92$ seconds.

Another particularity of the experimental setup presented in the main text is that, as the catalyst-loaded gels are quite small (2 mm of diameter at most), the consumption of the reactants in each micro-reactor is quite fast and we observe a decay in the periods of around 1-2 seconds every ten minutes. This is reported in Figure S2, where we observe a linear decay of the oscillation periods that can be fitted through linear regression to obtain a slope of $m = -0.002$. Depending on the initial period of the oscillations, that can range between 50 and 70 seconds (depending on room humidity and temperature), the mean life of the experiment could run between 2 to 4 hours, until the periods had decayed to $T = 35s$. From this point forward, spirals and other irregular behaviors appeared in the system. Thus, we performed our control experiments regularly in the period window between $T \in (40, 60)$ seconds.

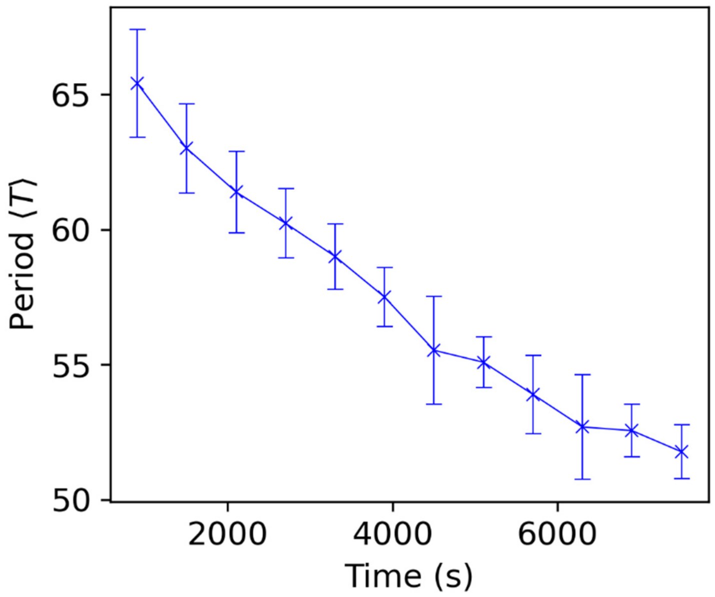

Figure S2: Decay of the oscillation periods in time. A linear regression to this data returns a slope of $m = -0.002$, revealing a decay of 1.2s every 10 minutes.

In some of the experiments conducted with the directed star network configuration it was possible to observe other outputs different from the global synchronization presented in the main text. In particular, for a small fraction of the experiments clusterization was observed as shown in Figure 3S. Here, the set of oscillators splits in two groups that oscillate with the same period but divided in two clusters with different phases (grouped with a black and red box in figure S3). Oscillators within the red rectangle are clearly synchronized although those in the black rectangle exhibit a larger dispersion in phase. There is no clear evidence of the mechanism that triggers clustering of global synchronization although our preliminary analysis indicates that it is related with the initial distribution of oscillator's periods.

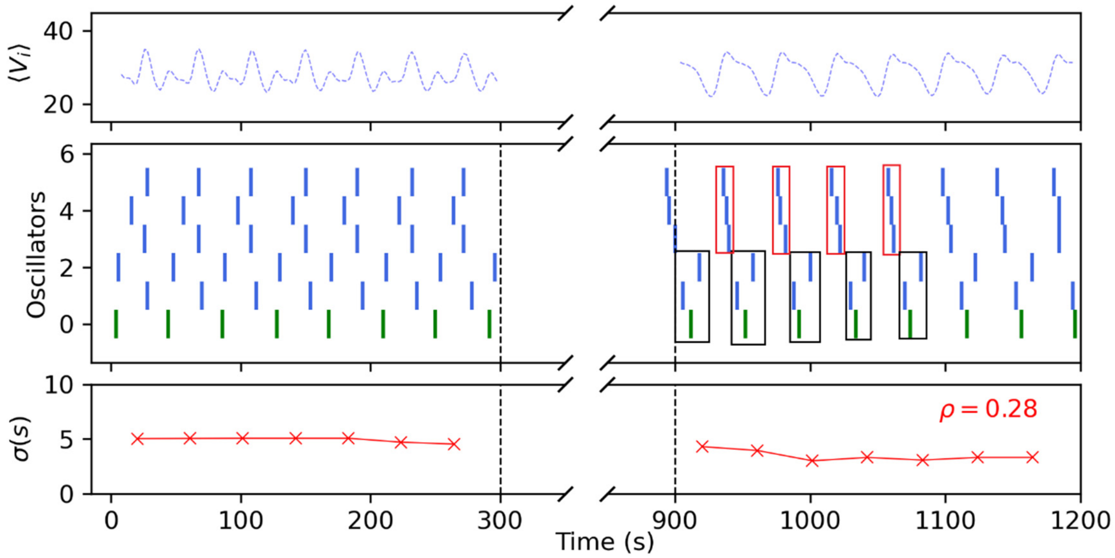

Figure S3: Network experiment where two clusters at different phases but similar frequency can be appreciated highlighted with black and red squares.
